# Supplementary material for: Chronic systemic inflammation predicts long-term mortality among patients with fatty liver disease: Data from the National Health and Nutrition Examination Survey 2007–2018
Source: PLoS One. 2024 Nov 18;19(11):e0312877. doi: 10.1371/journal.pone.0312877 (PMC11573152; doi:10.1371/journal.pone.0312877)
Supplement: S1 Table — (DOCX) [file pone.0312877.s001.docx]

**Table S1**. Baseline characteristics of patients according to SIRI tertile.

| **Variable** | **≤ 1437.3** | **1437.3 ~ 2179.1** | **≥ 2179.1** | ***P*-value** |
| --- | --- | --- | --- | --- |
| Age, years | 52.65 (0.47) | 49.99 (0.45) | 46.68 (0.52) | **< 0.0001** |
| Sex |  |  |  | **< 0.0001** |
| Female | 557 (26.89) | 811 (41.89) | 1175 (63.92) |  |
| Male | 1275 (73.11) | 1021 (58.11) | 658 (36.08) |  |
| Ethnicity |  |  |  | **< 0.0001** |
| Mexican American | 298 (8.55) | 372 (11.64) | 410 (13.14) |  |
| Non-Hispanic Black | 406 (11.09) | 305 (8.48) | 341 (10.50) |  |
| Non-Hispanic White | 822 (70.66) | 802 (69.21) | 716 (62.95) |  |
| Other Hispanic | 196 (5.30) | 229 (5.96) | 223 (7.20) |  |
| Other races | 110 (4.40) | 124 (4.70) | 143 (6.21) |  |
| Marital status |  |  |  | 0.41 |
| No | 683 (33.36) | 642 (31.88) | 740 (34.42) |  |
| Yes | 1149 (66.64) | 1190 (68.12) | 1093 (65.58) |  |
| Education |  |  |  | 0.4 |
| < high school | 771 (40.15) | 798 (39.98) | 755 (38.92) |  |
| high school | 722 (35.37) | 698 (34.71) | 753 (38.46) |  |
| > high school | 339 (24.48) | 336 (25.31) | 325 (22.62) |  |
| BMI, kg/m^2^ | 33.24 (0.18) | 34.07 (0.20) | 35.73 (0.23) | **< 0.0001** |
| PIR | 3.07 (0.06) | 2.91 (0.06) | 2.71 (0.07) | **< 0.0001** |
| WC, cm | 112.04 (0.41) | 112.80 (0.43) | 114.27 (0.46) | **0.001** |
| ALT, U/L | 33.06 (0.74) | 31.20 (0.52) | 27.51 (0.55) | **< 0.0001** |
| AST, U/L | 29.51 (0.54) | 26.79 (0.46) | 25.17 (0.54) | **< 0.0001** |
| Total cholesterol, mmol/L | 5.13 (0.04) | 5.12 (0.03) | 5.14 (0.04) | 0.86 |
| HDL-cholesterol, mmol/L | 1.21 (0.01) | 1.20 (0.01) | 1.22 (0.01) | 0.41 |
| SII | 349.68 (4.45) | 514.95 (6.06) | 754.13 (9.33) | **< 0.0001** |
| PIV | 226.52 (4.74) | 306.48 (5.76) | 401.10 (8.25) | **< 0.0001** |
| SIRI | 1096.48 (7.05) | 1778.87 (7.40) | 3143.65 (30.84) | **< 0.0001** |
| Drinking |  |  |  | **< 0.001** |
| Never | 192 (8.68) | 239 (12.29) | 247 (11.64) |  |
| Former | 322 (16.26) | 316 (16.52) | 323 (17.94) |  |
| Mild | 585 (38.14) | 537 (35.48) | 452 (30.35) |  |
| Moderate | 185 (12.43) | 222 (15.85) | 264 (17.50) |  |
| Heavy | 404 (24.49) | 344 (19.86) | 388 (22.57) |  |
| Smoking |  |  |  | **< 0.0001** |
| No | 914 (50.16) | 994 (54.39) | 953 (51.19) |  |
| Former | 603 (33.76) | 510 (29.33) | 436 (24.64) |  |
| Now | 294 (14.87) | 345 (18.08) | 448 (22.86) |  |
| Physical work |  |  |  | **0.04** |
| No | 1024 (50.27) | 1069 (53.07) | 1084 (55.68) |  |
| Mild | 390 (24.68) | 389 (23.86) | 416 (25.35) |  |
| Medium to high | 418 (25.05) | 374 (23.07) | 333 (18.97) |  |
| Hypertension |  |  |  | 0.13 |
| No | 772 (45.31) | 853 (49.08) | 876 (49.72) |  |
| Yes | 1060 (54.69) | 979 (50.92) | 957 (50.28) |  |
| Diabetes |  |  |  | 0.23 |
| No | 1248 (74.68) | 1250 (72.88) | 1212 (71.28) |  |
| Yes | 584 (25.32) | 582 (27.12) | 621 (28.72) |  |

Abbreviations: BMI, body mass index; WC, waist circumference; PIR, family income-to-poverty ratio; ALT, alanine transaminase; AST, aspartate transaminase, HDL, high-density lipoprotein; SII, systemic immune-inflammation index; PIV, pan-immune-inflammation value; SIRI, systemic inflammation response index. Continuous variables were described as mean and standard deviation. Categorical variables were expressed as numbers (percentages).
