# Supplementary material for: Chronic systemic inflammation predicts long-term mortality among patients with fatty liver disease: Data from the National Health and Nutrition Examination Survey 2007–2018
Source: PLoS One. 2024 Nov 18;19(11):e0312877. doi: 10.1371/journal.pone.0312877 (PMC11573152; doi:10.1371/journal.pone.0312877)
Supplement: S2 Table — (DOCX) [file pone.0312877.s002.docx]

**Table S2**. Univariate analysis for all-cause mortality and cardiovascular mortality.

| Character | Coef | Se (coef) | Robust se | Z | Pr(>\|z\|) | HR (95% CI) |
| --- | --- | --- | --- | --- | --- | --- |
| All-cause mortality | | | | | | |
| Age, years | 0.0955 | 0.0047 | 0.0053 | 17.9761 | **<0.0001** | 1.1003 (1.0889, 1.1118) |
| Sex |  |  |  |  |  |  |
| Female | Ref | Ref | Ref | Ref | Ref | Ref |
| Male | 0.2975 | 0.1124 | 0.1304 | 2.2806 | **0.0226** | 1.3465 (1.0427,  1.7388) |
| Ethnicity |  |  |  |  |  |  |
| Mexican American | Ref | Ref | Ref | Ref | Ref | Ref |
| Non-Hispanic Black | 0.7045 | 0.2927 | 0.1984 | 3.5503 | **<0.001** | 2.0228 (1.3710,  2.9844) |
| Non-Hispanic White | 0.8569 | 0.2454 | 0.2033 | 4.2162 | **<0.0001** | 2.3560 (1.5818,  3.5089) |
| Other Hispanic | 0.0815 | 0.3898 | 0.2566 | 0.3175 | 0.7509 | 1.0849 (0.6561,  1.7940) |
| Other races | 0.7315 | 0.3472 | 0.381 | 1.92 | 0.0549 | 2.0783 (0.9849,  4.3855) |
| Marital status |  |  |  |  |  |  |
| No | Ref | Ref | Ref | Ref | Ref | Ref |
| Yes | -0.2321 | 0.1114 | 0.101 | -2.2979 | **0.0216** | 0.7929 (0.6505, 0.9665) |
| Education |  |  |  |  |  |  |
| < high school | Ref | Ref | Ref | Ref | Ref | Ref |
| high school | 0.0453 | 0.1173 | 0.101 | 0.4482 | 0.654 | 1.0463 (0.8583,  1.2755) |
| > high school | -0.5523 | 0.1645 | 0.223 | -2.4771 | **0.0132** | 0.5756 (0.3718, 0.8911) |
| BMI, kg/m^2^ | -0.0289 | 0.0101 | 0.0131 | -2.2038 | **0.0275** | 0.9715 (0.9468, 0.9968) |
| PIR | -0.2156 | 0.0356 | 0.0409 | -5.2685 | **<0.0001** | 0.8061 (0.7440, 0.8734) |
| WC, cm | 0.0141 | 0.004 | 0.0044 | 3.1838 | **0.0015** | 1.0142 (1.0054, 1.0230) |
| ALT, U/L | -0.017 | 0.0041 | 0.0081 | -2.1005 | **0.0357** | 0.9832 (0.9678, 0.9989) |
| AST, U/L | 0.0016 | 0.0015 | 0.001 | 1.5308 | 0.1258 | 1.0016 (0.9996, 1.0036) |
| Total cholesterol, mmol/L | -0.3682 | 0.0551 | 0.0555 | -6.6382 | **<0.0001** | 0.6920 (0.6207, 0.7715) |
| HDL-cholesterol, mmol/L | 0.0432 | 0.1716 | 0.1906 | 0.2264 | 0.8209 | 1.0441 (0.7186, 1.5171) |
| SII | 8.00E-04 | 1.00E-04 | 1.00E-04 | 5.4311 | **<0.0001** | 1.0008 (1.0005, 1.0011) |
| PIV | 8.00E-04 | 1.00E-04 | 1.00E-04 | 5.2511 | **<0.0001** | 1.0008 (1.0005, 1.0011) |
| SIRI | -1.00E-04 | 1.00E-04 | 1.00E-04 | -1.344 | 0.1789 | 0.9999 (0.9998, 1.0000) |
| Drinking |  |  |  |  |  |  |
| Never | Ref | Ref | Ref | Ref | Ref | Ref |
| Former | 0.4972 | 0.1792 | 0.2024 | 2.4569 | **0.014** | 1.6442 (1.1058, 2.4446) |
| Mild | -0.4135 | 0.1871 | 0.1985 | -2.0831 | **0.0372** | 0.6613 (0.4482, 0.9759) |
| Moderate | -0.8173 | 0.2474 | 0.3487 | -2.3441 | **0.0191** | 0.4416 (0.2230, 0.8746) |
| Heavy | -0.8008 | 0.2197 | 0.2323 | -3.4468 | **<0.001** | 0.449 (0.2848, 0.7079) |
| Smoking |  |  |  |  |  |  |
| No | Ref | Ref | Ref | Ref | Ref | Ref |
| Former | 0.7183 | 0.1222 | 0.1581 | 4.5428 | **<0.0001** | 2.051 (1.5044, 2.7961) |
| Now | 0.449 | 0.1475 | 0.1566 | 2.8662 | **0.0042** | 1.5667 (1.1525, 2.1297) |
| Physical work |  |  |  |  |  |  |
| No | Ref | Ref | Ref | Ref | Ref | Ref |
| Mild | -0.5663 | 0.1421 | 0.1246 | -4.5435 | **<0.0001** | 0.5677 (0.4446, 0.7247) |
| Medium to high | -0.8419 | 0.1688 | 0.185 | -4.5514 | **<0.0001** | 0.4309 (0.2999, 0.6192) |
| Hypertension |  |  |  |  |  |  |
| No | Ref | Ref | Ref | Ref | Ref | Ref |
| Yes | 1.2097 | 0.1294 | 0.1451 | 8.3364 | **<0.0001** | 3.3524 (2.5225, 4.4552) |
| Diabetes |  |  |  |  |  |  |
| No | Ref | Ref | Ref | Ref | Ref | Ref |
| Yes | 1.2927 | 0.1089 | 0.1391 | 9.2953 | **<0.0001** | 3.6427 (2.7736, 4.7841) |
| Cardiovascular mortality | | | | | | |
| Age, years | 0.1149 | 0.0136 | 0.0155 | 7.4238 | **<0.0001** | 1.1218 (1.0883, 1.1564) |
| Sex |  |  |  |  |  |  |
| Female | Ref | Ref | Ref | Ref | Ref | Ref |
| Male | -0.042 | 0.284 | 0.2435 | -0.1724 | 0.8632 | 0.9589 (0.5950, 1.5453) |
| Ethnicity |  |  |  |  |  |  |
| Mexican American | Ref | Ref | Ref | Ref | Ref | Ref |
| Non-Hispanic Black | 0.7922 | 0.6922 | 0.4335 | 1.8276 | 0.0676 | 2.2083 (0.9442, 5.1644) |
| Non-Hispanic White | 0.5298 | 0.5946 | 0.402 | 1.3181 | 0.1875 | 1.6987 (0.7726, 3.7348) |
| Other Hispanic | 0.3424 | 0.8645 | 0.57 | 0.6008 | 0.548 | 1.4084 (0.4608, 4.3041) |
| Other races | 1.3937 | 0.7147 | 0.7785 | 1.7901 | 0.0734 | 4.0295 (0.8761,  18.5325) |
| Marital status |  |  |  |  |  |  |
| No | Ref | Ref | Ref | Ref | Ref | Ref |
| Yes | -0.3853 | 0.2862 | 0.3136 | -1.2286 | 0.2192 | 0.6803 (0.3679, 1.2578) |
| Education |  |  |  |  |  |  |
| < high school | Ref | Ref | Ref | Ref | Ref | Ref |
| high school | 0.2707 | 0.3086 | 0.2839 | 0.9537 | 0.3403 | 1.3109 (0.7515,  2.2867) |
| > high school | -0.4238 | 0.4357 | 0.5158 | -0.8215 | 0.4113 | 0.6546 (0.2382,  1.7990) |
| BMI, kg/m^2^ | -0.0111 | 0.0249 | 0.0233 | -0.4747 | 0.635 | 0.989 (0.9449,  1.0352) |
| PIR | -0.3256 | 0.099 | 0.1035 | -3.1458 | **0.0017** | 0.7221 (0.5895,  0.8845) |
| WC, cm | 0.0187 | 0.0101 | 0.0096 | 1.9503 | 0.0511 | 1.0189 (0.9999,  1.0382) |
| ALT, U/L | -0.0362 | 0.014 | 0.0236 | -1.5384 | 0.124 | 0.9644 (0.9209,  1.0100) |
| AST, U/L | -0.0011 | 0.0083 | 0.0087 | -0.1234 | 0.9018 | 0.9989 (0.9820,  1.0161) |
| Total cholesterol, mmol/L | -0.372 | 0.1435 | 0.1494 | -2.4907 | **0.0127** | 0.6893 (0.5144,  0.9238) |
| HDL-cholesterol, mmol/L | 1.01 | 0.3669 | 0.3429 | 2.9451 | **0.0032** | 2.7456 (1.4019,  5.3771) |
| SII | 9.00E-04 | 3.00E-04 | 2.00E-04 | 4.5703 | **<0.0001** | 1.0009 (1.0005,  1.0012) |
| PIV | 8.00E-04 | 2.00E-04 | 2.00E-04 | 4.8409 | **<0.0001** | 1.0008 (1.0005,  1.0011) |
| SIRI | 1.00E-04 | 1.00E-04 | 1.00E-04 | 0.5344 | 0.5931 | 1.0001 (0.9998,  1.0003) |
| Drinking |  |  |  |  |  |  |
| Never | Ref | Ref | Ref | Ref | Ref | Ref |
| Former | 0.4572 | 0.4824 | 0.4785 | 0.9554 | 0.3394 | 1.5796 (0.6183, 4.0355) |
| Mild | -0.3658 | 0.4968 | 0.4957 | -0.738 | 0.4605 | 0.6936 (0.2625, 1.8327) |
| Moderate | -0.6925 | 0.6354 | 0.6328 | -1.0944 | 0.2738 | 0.5003 (0.1447,  1.7294) |
| Heavy | -1.0771 | 0.631 | 0.5526 | -1.9492 | 0.0513 | 0.3406 (0.1153, 1.0059) |
| Smoking |  |  |  |  |  |  |
| No | Ref | Ref | Ref | Ref | Ref | Ref |
| Former | 0.6001 | 0.2958 | 0.3407 | 1.7614 | 0.0782 | 1.8223 (0.9346, 3.5531) |
| Now | -0.5993 | 0.5122 | 0.4138 | -1.4482 | 0.1475 | 0.5492 (0.2441, 1.2358) |
| Physical work |  |  |  |  |  |  |
| No | Ref | Ref | Ref | Ref | Ref | Ref |
| Mild | -0.4415 | 0.3508 | 0.3403 | -1.2974 | 0.1945 | 0.643 (0.3300,  1.2529) |
| Medium to high | -1.0011 | 0.47 | 0.4626 | -2.164 | **0.0305** | 0.3675 (0.1484, 0.9099) |
| Hypertension |  |  |  |  |  |  |
| No | Ref | Ref | Ref | Ref | Ref | Ref |
| Yes | 2.1644 | 0.4619 | 0.3662 | 5.9112 | **<0.0001** | 8.7093 (4.2493,  17.8506) |
| Diabetes |  |  |  |  |  |  |
| No | Ref | Ref | Ref | Ref | Ref | Ref |
| Yes | 1.6329 | 0.2918 | 0.3337 | 4.8938 | **<0.0001** | 5.1186 (2.6616, 9.8440) |

Abbreviations: Coef, coefficient; se, standard error; HR, hazard ratio; 95% CI, 95% confidence interval; Ref, reference; BMI, body mass index; WC, waist circumference; PIR, family income-to-poverty ratio; ALT, alanine transaminase; AST, aspartate transaminase, HDL, high-density lipoprotein; SII, systemic immune-inflammation index; PIV, pan-immune-inflammation value; SIRI, systemic inflammation response index.
