# Supplementary material for: Chronic systemic inflammation predicts long-term mortality among patients with fatty liver disease: Data from the National Health and Nutrition Examination Survey 2007–2018
Source: PLoS One. 2024 Nov 18;19(11):e0312877. doi: 10.1371/journal.pone.0312877 (PMC11573152; doi:10.1371/journal.pone.0312877)
Supplement: S3 Table — (DOCX) [file pone.0312877.s003.docx]

**Table S3**. Multivariate Cox models for SII and PIV and cardiovascular mortality.

|  | Crude Model HR (95%CI), *P*-value | Fully adjusted Model HR (95%CI), *P*-value |
| --- | --- | --- |
| SII (continuous) (mortality incidence: 75/5497) | 1.0006 (1.0002, 1.0011), **0.0087** | 1.0006 (1.0001, 1.0011), **0.0293** |
| SII tertile |  |  |
| T1 (19/1832) | 1.0 | 1.0 |
| T2 (21/1832) | 1.0609 (0.5703, 1.9738), 0.8519 | 1.1194 (0.5635, 2.2238), 0.7473 |
| T3 (35/1833) | 1.7620 (1.0074, 3.0820), **0.0471** | 1.7462 (0.9308, 3.2757), 0.0824 |
| PIV (continuous) (mortality incidence: 75/5497) | 1.0006 (1.0002, 1.0010), **0.0040** | 1.0007 (1.0002, 1.0013), **0.0076** |
| PIV tertile |  |  |
| T1 (15/1832) | 1.0 | 1.0 |
| T2 (27/1832) | 1.8207 (0.9683, 3.4236), 0.0629 | 1.8704 (0.9189, 3.8072), 0.0842 |
| T3 (33/1833) | 2.3110 (1.2545, 4.2575), **0.0072** | 2.1350 (1.0729, 4.2485), **0.0307** |

The crude Model was a crude model with no adjustment, and the fully adjusted model adjusted for all significant covariates in the univariate analysis including age, PIR, total cholesterol, HDL- cholesterol, physical work, hypertension, and diabetes. Abbreviations: SII, systemic immune-inflammation index; PIV, pan-immune-inflammation value; PIR, family income-to-poverty ratio; HR, hazard ratio; 95% CI, 95% confidence interval.
