# Supplementary material for: Chronic systemic inflammation predicts long-term mortality among patients with fatty liver disease: Data from the National Health and Nutrition Examination Survey 2007–2018
Source: PLoS One. 2024 Nov 18;19(11):e0312877. doi: 10.1371/journal.pone.0312877 (PMC11573152; doi:10.1371/journal.pone.0312877)
Supplement: S4 Table — (DOCX) [file pone.0312877.s004.docx]

**Table S4**. Association of CRP with all-cause and CVD mortality in patients with FLD, NHANES 2007-2018.

|  | Fully adjusted Model HR (95%CI), P-value |
| --- | --- |
| **ALL-cause** |  |
| CRP | 1.089 (1.008, 1.177) 0.0314 |
| CRP quartile |  |
| Q1 | 1.0 |
| Q2 | 0.765 (0.552, 1.061) 0.1085 |
| Q3 | 0.842 (0.604, 1.174) 0.3101 |
| Q4 | 1.317 (1.063, 1.802) 0.0002 |
| P for trend | 0.0426 |
| **CVD** |  |
| CRP | 1.122 (0.992, 1.270) 0.0360 |
| CRP quartile |  |
| Q1 | 1.0 |
| Q2 | 0.570 (0.314, 1.035) 0.0648 |
| Q3 | 0.895 (0.514, 1.560) 0.6962 |
| Q4 | 1.376 (1.108, 2.342) 0.0093 |
| P for trend | 0.0219 |

For all-cause mortality, the fully adjusted model was adjusted for all important covariates in the univariate analysis including age, BMI, PIR, sex, ethnicity, marital status, education level, WC, ALT, total cholesterol, smoking, alcohol consumption, physical work, hypertension, and diabetes. For cardiovascular mortality, the fully adjusted model was adjusted for all significant covariates including age, PIR, total cholesterol, HDL- cholesterol, physical work, hypertension, and diabetes.
