# Supplementary material for: Chronic systemic inflammation predicts long-term mortality among patients with fatty liver disease: Data from the National Health and Nutrition Examination Survey 2007–2018
Source: PLoS One. 2024 Nov 18;19(11):e0312877. doi: 10.1371/journal.pone.0312877 (PMC11573152; doi:10.1371/journal.pone.0312877)
Supplement: S5 Table — (DOCX) [file pone.0312877.s005.docx]

**Table S5**. Correlation analysis of SII/PIV with CRP.

| **CRP** | Fully adjusted Model  β(95%CI) |
| --- | --- |
| **SII** | 0.001 (0.001, 0.001) <0.00001 |
| **SII** |  |
| T1 | Ref. |
| T2 | 0.054 (-0.026, 0.133) 0.18881 |
| T3 | 0.351 (0.271, 0.430) <0.00001 |
| **PIV** | 0.001 (0.001, 0.001) <0.00001 |
| **PIV** |  |
| T1 | Ref. |
| T2 | 0.071 (-0.008, 0.151) 0.07836 |
| T3 | 0.314 (0.234, 0.395) <0.00001 |

This fully adjusted model adjusted for all covariates except SII/PIV/SIRI and death-related variables.
