# Supplementary material for: Chronic systemic inflammation predicts long-term mortality among patients with fatty liver disease: Data from the National Health and Nutrition Examination Survey 2007–2018
Source: PLoS One. 2024 Nov 18;19(11):e0312877. doi: 10.1371/journal.pone.0312877 (PMC11573152; doi:10.1371/journal.pone.0312877)
Supplement: S7 Table — (DOCX) [file pone.0312877.s007.docx]

**Table S7**. Sensitivity analyses of the association of SII/PIV with all-cause mortality in patients with FLD (excluding those with <24 months of follow-up and extreme values of SI markers).

| **ALL** | **Fully adjusted Model HR (95%CI)** | **p** |
| --- | --- | --- |
| **SII** | 1.0006(1.0003,1.0009) | <0.001 |
| **SII** |  |  |
| T1 | ref | ref |
| T2 | 0.7468(0.5508,1.0126) | 0.0602 |
| T3 | 1.2792(0.8846,1.8499) | 0.1907 |
| **PIV** | 1.0009(1.0005,1.0013) | **<0.0001** |
| **PIV** |  |  |
| T1 | ref | ref |
| T2 | 1.1202(0.8002,1.5681) | 0.5085 |
| T3 | 1.4029(0.9815,2.0053) | 0.0632 |

The fully adjusted model was adjusted for all important covariates in the univariate analysis including age, BMI, PIR, sex, ethnicity, marital status, education level, WC, ALT, total cholesterol, smoking, alcohol consumption, physical work, hypertension, and diabetes.
