# Supplementary material for: Chronic systemic inflammation predicts long-term mortality among patients with fatty liver disease: Data from the National Health and Nutrition Examination Survey 2007–2018
Source: PLoS One. 2024 Nov 18;19(11):e0312877. doi: 10.1371/journal.pone.0312877 (PMC11573152; doi:10.1371/journal.pone.0312877)
Supplement: S8 Table — (DOCX) [file pone.0312877.s008.docx]

**Table S8**. Sensitivity analyses of the association of SII/PIV with CVD mortality in patients with FLD (excluding those with <24 months of follow-up and extreme values of SI markers).

| **CVD** | **Fully adjusted model**  **HR (95%CI)** | **p** |
| --- | --- | --- |
| **SII** | 1.0009(1.0006,1.0013) | **<0.0001** |
| **SII** |  |  |
| T1 | ref | ref |
| T2 | 0.5549(0.3478,0.8852) | **0.0135** |
| T3 | 1.6423(1.0142,2.6596) | **0.0437** |
| **PIV** | 1.0011(1.0008,1.0014) | **<0.0001** |
| **PIV** |  |  |
| T1 | ref | ref |
| T2 | 0.9571(0.5238,1.7490) | 0.8867 |
| T3 | 2.2793(1.3660,3.8032) | **0.0016** |

The fully adjusted model was adjusted for all significant covariates including age, PIR, total cholesterol, HDL- cholesterol, physical work, hypertension, and diabetes.
