# Supplementary material for: Chronic systemic inflammation predicts long-term mortality among patients with fatty liver disease: Data from the National Health and Nutrition Examination Survey 2007–2018
Source: PLoS One. 2024 Nov 18;19(11):e0312877. doi: 10.1371/journal.pone.0312877 (PMC11573152; doi:10.1371/journal.pone.0312877)
Supplement: S1 Fig — RCS models of SII (A) and PIV (B) with cardiovascular mortality in patients with FLD, NHANES 2007–2018. The horizontal axis of the RCS plot represents the level of SII/PIV, and the vertical axis represents the log hazard value for CVD mortality. (DOCX) [file pone.0312877.s009.docx]

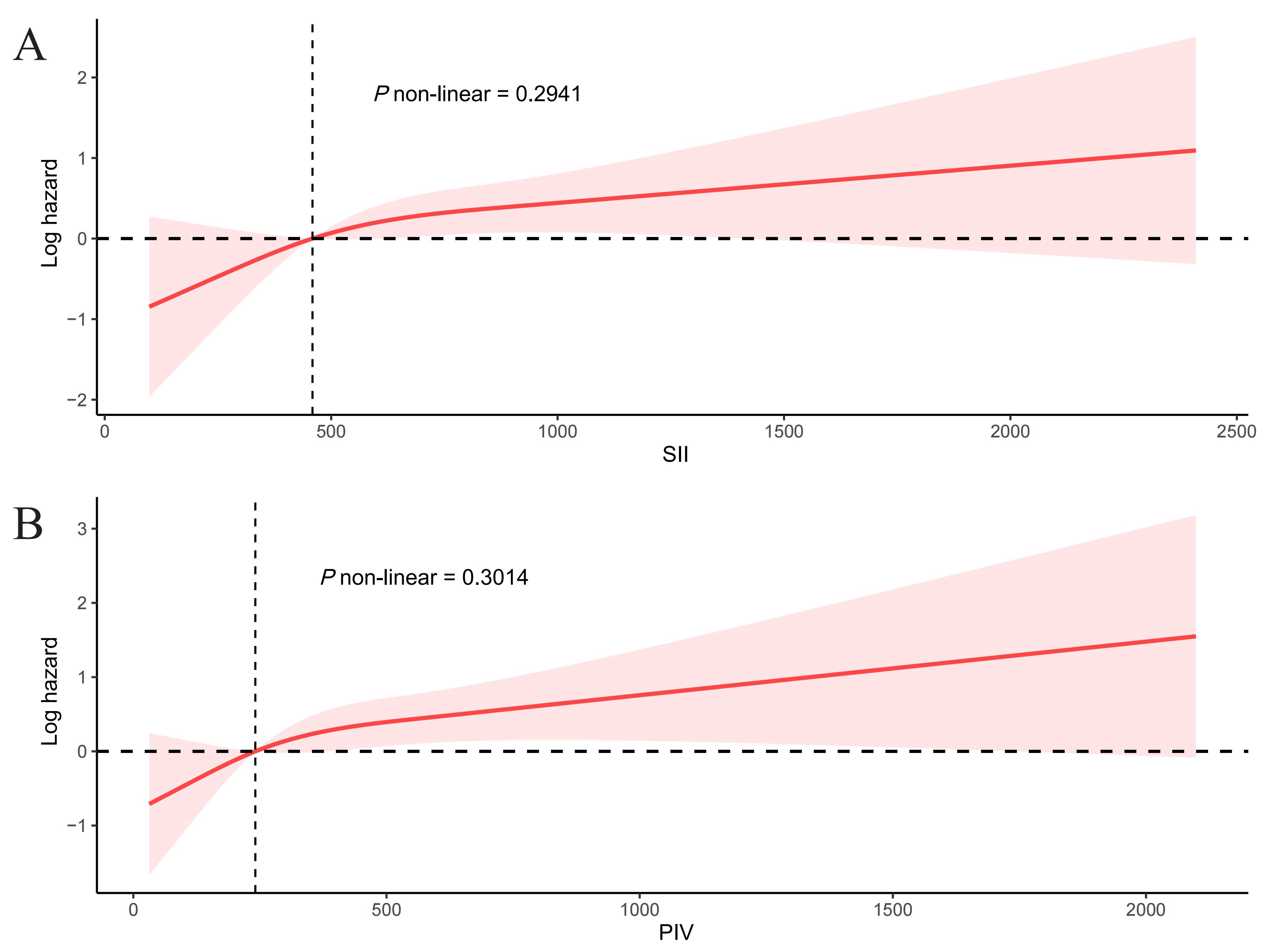


**Figure S1**. RCS models of SII (A) and PIV (B) with cardiovascular mortality. Abbreviations: RCS, restricted cubic spline; SII, systemic immune-inflammation index; PIV, pan-immune-inflammation value.
