# Supplementary material for: Chronic systemic inflammation predicts long-term mortality among patients with fatty liver disease: Data from the National Health and Nutrition Examination Survey 2007–2018
Source: PLoS One. 2024 Nov 18;19(11):e0312877. doi: 10.1371/journal.pone.0312877 (PMC11573152; doi:10.1371/journal.pone.0312877)
Supplement: S2 Fig — Subgroup analysis of SII (A) and PIV (B) with cardiovascular mortality in patients with FLD, NHANES 2007–2018. (DOCX) [file pone.0312877.s010.docx]

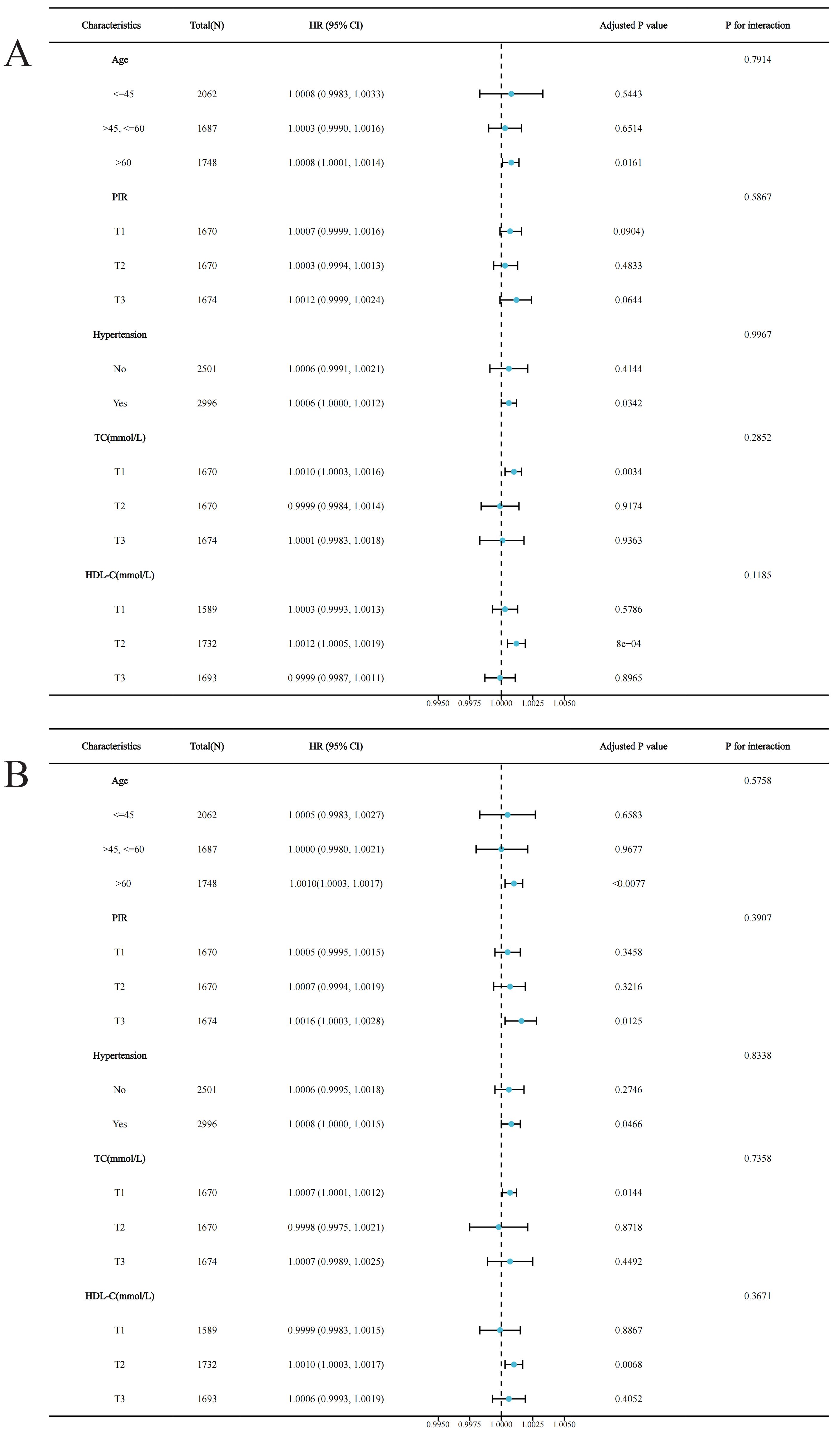


**Figure S2**. Subgroup analysis of SII (A) and PIV (B) with cardiovascular mortality. Abbreviations: SII, systemic immune-inflammation index; PIV, pan-immune-inflammation value; N, number; HR, hazard ratio; 95% CI, 95% confidence interval; PIR, family income-to-poverty ratio; TC, total cholesterol; HDL-C, high-density lipoprotein-cholesterol; T1, T2, T3 = tertile1, tertile2, tertile3.
