# Supplementary material for: Chronic systemic inflammation predicts long-term mortality among patients with fatty liver disease: Data from the National Health and Nutrition Examination Survey 2007–2018
Source: PLoS One. 2024 Nov 18;19(11):e0312877. doi: 10.1371/journal.pone.0312877 (PMC11573152; doi:10.1371/journal.pone.0312877)
Supplement: Attachment — Submitted filename: Response to reviewers.docx [file pone.0312877.s012.docx]

Dear Editors and Reviewers,

Thank you very much for your great efforts and patience in reviewing the manuscript and for giving us the opportunity to make revisions. We have responded to all the reviewers' comments and revised the manuscript accordingly. We believe that the quality of our manuscript will be significantly improved after the revision. The following is our response.

**Response to reviewer 1**

Major comments:
1. Section “Abstract”: Please provide more details regarding the design (in brief) of the NHANES cohort and on the collection of follow-up information. Moreover, the description of the statistical methods used and the presentation of results used appears confusing and therefore should be carefully revised. For example, since Cox proportional hazard models are frequently used for time-to event analyses, including survival analysis, the difference between Cox models and survival analyses remains unclear based on the information in the abstract.

**RESPONSE**: We appreciate your constructive and careful suggestions. We strongly agree with you and have added more details of NHANES' cohort design to the abstract as follows: “Baseline FLD participants from NHANES 2007-2018 were follow-up until December 31, 2019, and were prospectively matched with records in the National Death Index database to obtain mortality information.” In addition, we have revised the description of the statistical analyses in the abstract to address the ambiguous portions in response to your comments, and the revised counterparts are as follows: “Univariate and multivariate Cox proportional hazards models were used to estimate hazard ratios for mortality outcomes. Kaplan-Meier (KM) analysis was used to estimate survival probabilities, and restricted cubic splines (RCS) were employed to explore potential nonlinear associations. Stratified analysis was conducted to assess the robustness of the findings across different subgroups.”

2.Section “Methods”: The description of the NHANES cohort should be improved, to allow a better understanding of the population/eligibility criteria in NHANES. Please also cite corresponding references regarding the study design of the NHANSE cohort for some guidance.

**RESPONSE:** We appreciate your constructive and careful suggestions. We have supplemented the description of the NHANES cohort in the Methods section with citations to the corresponding literature based on your comments, as follows: “NHANES is a nationally representative, multistage, cross-sectional survey, which is a major program of the National Center for Health Statistics (NCHS) and aimed to assess the health and nutrition status of US non-institutionalized populations. The NCHS Ethics Review Board has approved all NHANES study protocols, and all participants signed a written consent form. We adopted data from NHANES 2007-2018 (including 6 continuous cycles, 2007-2008, 2009-2010, 2011-2012, 2013-2014, 2015-2016, 2017-2018) for this study. Baseline FLD participants from NHANES 2007-2018 were followed until December 31, 2019, and mortality information was obtained by prospectively matching with death certificate information from the Public-use Linked Mortality Files of the National Death Index (NDI) database (https://www.cdc.gov/nchs/data-linkage/mortality-public.htm)[13, 18].”

References

13. Xia Y, Xia C, Wu L, Li Z, Li H, Zhang J. Systemic Immune Inflammation Index (SII), System Inflammation Response Index (SIRI) and Risk of All-Cause Mortality and Cardiovascular Mortality: A 20-Year Follow-Up Cohort Study of 42,875 US Adults. J Clin Med. 2023;12(3). doi: 10.3390/jcm12031128.

18. Liu Y, Geng T, Wan Z, Lu Q, Zhang X, Qiu Z, et al. Associations of Serum Folate and Vitamin B12 Levels With Cardiovascular Disease Mortality Among Patients With Type 2 Diabetes. JAMA Netw Open. 2022;5(1):e2146124. doi: 10.1001/jamanetworkopen.2021.46124.

3. Section “Methods”: The methods are very hard to understand. The section should be substantially revised to provide a clear understand of the methods used.

**RESPONSE**: We appreciate your constructive and detailed suggestions regarding the clarity of the Methods section. Based on your comments, we have made substantial revisions to improve the logical flow and structure of this section. Below are the specific modifications:**Participants Section**:We have added more context on the design of NHANES, including the fact that it is a nationally representative, multistage, cross-sectional survey led by the National Center for Health Statistics (NCHS). This clarifies the nature of the population sampled and the methodology behind NHANES.We provided a detailed explanation of the exclusion criteria used to derive the final sample of 5,497 participants. This includes the removal of individuals without FLD diagnostic indicators, those with missing mortality data, missing covariate information, and extreme values for systemic inflammation (SI) markers. These steps clarify how we arrived at the final population.We clarified the follow-up process, specifying that FLD participants were followed until December 31, 2019, and mortality information was obtained by matching with the National Death Index (NDI) database.**Definition of SI Markers:**We retained the original definitions of the SI markers (SII, SIRI, PIV) but added more detail regarding how they were calculated using data from complete blood counts (CBC). This makes it clear that the markers are derived from specific blood parameters.**Definition of FLD:**We retained the use of the Fatty Liver Index (FLI) to define hepatic steatosis and included a detailed breakdown of the formula used to calculate FLI. This ensures a clear understanding of how FLD was diagnosed in the study.**Outcome Ascertainment:**We clarified how mortality data was obtained, specifying that it was matched with death certificates in the NDI database and coded according to ICD-10 classifications for cardiovascular deaths. This ensures transparency in the outcome data collection process. **Covariate Determination:**We have provided a more detailed description of the key confounding factors used in the models, such as smoking, alcohol consumption, and physical activity, and how these factors were categorized. This step helps to better explain the variables adjusted for in our analysis.**Statistical Analysis:**We have restructured the statistical analysis section to clearly differentiate between univariate and multivariate Cox proportional hazards models. We now explicitly state which covariates were included in the fully adjusted models for both all-cause and cardiovascular mortality.We added a more detailed explanation of Kaplan-Meier survival curves and log-rank tests, and how these were used to estimate survival probabilities across different levels of SI markers. This further clarifies the survival analysis techniques used.We explained the use of restricted cubic splines (RCS) for exploring nonlinear relationships and described how the degrees of freedom (knots) were determined based on statistical significance. This addresses the more complex statistical analyses performed in the study.Stratified analysis and sensitivity analysis details were expanded, including how we tested the stability of our results across different subgroups and how other inflammatory markers, such as C-reactive protein (CRP), were analyzed in relation to SII and PIV. This provides a more complete understanding of the robustness checks we performed.We added a detailed description of the sensitivity analyses, particularly focusing on the inclusion of participants with shorter follow-up times (<24 months) and extreme SI values to assess the robustness of the results.We explained the threshold effect analysis of SII on all-cause mortality, which explores the nonlinear association and potential cutoff points for increased mortality risk. This further strengthens our findings.

4.Section “Methods”: While the use of stepwise models may be of advantage for co-variate adjustment in datasets with small samples, stepwise regression models appear not ideal to analyze the associations between markers of inflammation and clinical outcomes in the present dataset. Cox proportional hazard models or semiparametric models should be preferred, the sample size allows for a comprehensive list of covariates without the need for any stepwise approach.

**RESPONSE:** We strongly agree with your comment that Cox proportional hazards models or semiparametric models are more suitable for analyzing the associations between markers of inflammation and clinical outcomes in our dataset, given the sample size and the comprehensive list of covariates.we have made the following changes:Removal of Stepwise Regression Models: We have removed the description of stepwise regression from the Methods section, as well as the stepwise multiple regression table in the Supplementary Material. We agree that the sample size allows us to include a full range of covariates in the Cox proportional hazards models without resorting to stepwise selection.Replacement with Cox Proportional Hazards Models: We now rely solely on Cox proportional hazards models for multivariable adjustment, ensuring a more robust and interpretable analysis. All covariates that were considered important based on prior literature and clinical relevance have been included in the models without any stepwise reduction.We appreciate your constructive and insightful suggestions regarding the use of stepwise regression models.

5.Section “Methods”: Please explain the choice of the covariates used for adjustment in model 2. It is unclear why a set of recognized predictors of adverse clinical events were not used. Please also list the co-variates used in model 3.

**RESPONSE:** We appreciate your constructive and careful suggestions. We couldn't agree more with your suggestion. For the selection of covariates for the previous partial adjustment of Model 2, we picked certain underlying covariates based on previous studies similarly using NHANES ((e.g. PMID: 36817427, doi:10.3389/fimmu.2023.1087345), i.e., demographic variables such as age. Thus, Model 2 was originally constructed to further adjust the underlying demographic variables based on the crude model in order to observe changes in effect sizes and to derive whether the association was independent of simple demographic variables. However, in light of your comments, we have deleted the original Model 2 and retained only the crude and fully adjusted models to more clearly reflect the process of adjusting for covariates and to reduce confusion about covariate selection. Finally, we have added covariates to Model 3 (fully adjusted model) at your request as follows: “For all-cause mortality, crude model was an unadjusted crude model, and the fully adjusted model was adjusted for all important covariates in the univariate analysis including age, BMI, PIR, sex, ethnicity, marital status, education level, WC, ALT, total cholesterol, smoking, alcohol consumption, physical work, hypertension, and diabetes. For cardiovascular mortality, crude model was a crude model, and the fully adjusted model was adjusted for all significant covariates including age, PIR, total cholesterol, HDL- cholesterol, physical work, hypertension, and diabetes.”

6. Section “Result”: The term “linked mortality data” in Table 1 is confusion. The risk for clinical outcomes according to indices of systemic inflammation should be presented in Tables 3+4 while providing incidence rates per 100-patient years in addition to the crude and adjusted HRs.

**RESPONSE**: We appreciate your constructive and careful suggestions. We have deleted the inappropriate descriptions in Table 1 and added descriptions of incidence rates to Tables 3/4 as requested.

**Table 3**. Multivariate adjusted Cox models for SII and all-cause mortality.

|  | Crude Model HR (95%CI) *P* | Fully adjusted Model HR (95%CI) *P* |
| --- | --- | --- |
| SII (continuous) (mortality incidence: 464/5497) | 1.0006 (1.0004, 1.0008), **< 0.0001** | 1.0004 (1.0002, 1.0006), **< 0.0001** |
| SII tertile |  |  |
| T1 (129/1832) | 1.0 | 1.0 |
| T2 (127/1832) | 0.9561 (0.7483, 1.2216), 0.7193 | 0.9012 (0.6888, 1.1789), 0.4477 |
| T3 (208/1833) | 1.5648 (1.2560, 1.9495), **< 0.0001** | 1.3268 (1.0398, 1.6930), **0.0230** |

Abbreviations: BMI, body mass index; PIR, family income-to-poverty ratio. The fully adjusted model was adjusted for all important covariates in the univariate analysis including age, BMI, PIR, sex, ethnicity, marital status, education level, WC, ALT, total cholesterol, smoking, alcohol consumption, physical work, hypertension, and diabetes.

**Table 4**. Multivariate adjusted Cox models for PIV and all-cause mortality.

|  | Crude Model HR (95%CI), *P*-value | Fully adjusted Model HR (95%CI), *P*-value |
| --- | --- | --- |
| PIV (continuous) (mortality incidence: 464/5497) | 1.0006 (1.0005, 1.0008), **< 0.0001** | 1.0006 (1.0003, 1.0008), **0.0002** |
| PIV tertile |  |  |
| T1 (111/1832) | 1.0 | 1.0 |
| T2 (146/1832) | 1.3407 (1.0473, 1.7161), **0.0200** | 1.2377 (0.9421, 1.6260), 0.1256 |
| T3 (207/1833) | 1.9742 (1.5674, 2.4867), **< 0.0001** | 1.3212 (1.0188, 1.7134), **0.0357** |

Abbreviations: BMI, body mass index; PIR, family income-to-poverty ratio. The fully adjusted model was adjusted for all important covariates in the univariate analysis including age, BMI, PIR, sex, ethnicity, marital status, education level, WC, ALT, total cholesterol, smoking, alcohol consumption, physical work, hypertension, and diabetes.

7. Section “Results”: Please structure the section based on the implications of systemic inflammation makers (e.g. Baseline characteristics, associations of different markers with clinical outcomes), not on the statistical method used. The findings for all associations should be described by all corresponding models (categorical, continuous, Poission, interaction), respectively.

**RESPONSE**: We appreciate your constructive and careful suggestions. We apologize for the distress caused by the inappropriate description. We have substantially revised the Methods section to make it clearer, as indicated in the Results section of the revised manuscript.

8. Section “Results”: The indices of systemic inflammation are based on counts of platelet/neutrophil/monocyte count/lymphocyte count. How do the findings relate to inflammatory biomarkers such as IL-6 or hsCRP? Please add the analyses of the associations of additional inflammatory biomarkers with clinical outcomes.

**RESPONSE**: We appreciate your constructive and careful suggestions. We strongly agree with your suggestion and have added the appropriate analyses. Since NHANES only has data on CRP and not on other inflammatory markers such as IL-6, we explored the association of CRP with mortality in patients with FLD and elucidated the correlation between SII/PIV and CRP.We added the corresponding sensitivity analysis section in the manuscript: “In sensitivity analyses, we first explored the association of other inflammatory markers such as C-reactive protein (CRP) with mortality in patients with FLD and the correlation of CRP with SII/PIV.”, “CRP was similarly positively associated with all-cause and CVD mortality in the FLD population (all-cause: HR 1.089, p=0.0314; CVD: HR 1.122, p=0.0360). Notably, there was also a significant positive correlation between SII/PIV and CRP (both β 0.001), suggesting that SII/PIV and other inflammatory markers, such as CRP, may significantly interact and collectively be associated with mortality in the FLD population (**Table S4 and S5**).”

Minor comments:
1. Section “Methods”: The paragraph summarizing the covariable selection can be streamlined.

**RESPONSE**: We appreciate your constructive and careful suggestions. We simplified the covariate selection section based on comments as follows: “We selected several important confounding factors including age, sex, ethnicity, education level, family income-to-poverty ratio (PIR), and marital status, smoking, alcohol consumption, physical work, BMI, WC, total cholesterol, high-density lipoprotein (HDL)-cholesterol, alanine transaminase (ALT), aspartate transaminase (AST), hypertension, and diabetes. We defined types of smoking as: (1) Never: individuals who answered that they had smoked less than 100 cigarettes in life; (2) Former: have smoked at least 100 cigarettes throughout their lives, but are currently not smoking; (3) Current: had smoked at least 100 cigarettes in life and smoked at present[24]. We determined types of drinking as: (1) Never: alcohol consumption < 12 times per lifetime; (2) Former: at least 12 drinking occasions within a year or lifetime and no drinking in the last year (3) Heavy: at least 3 drinks/d for women/4 drinks/d for men, or binge drinking ≥ 5 d/month; (4) Moderate: at least 2 drinks/d for women/3 drinks/d for men, or binge drinking ≥ 2 d/month; (5) Mild: less than 2 drinks/d[25]. For physical work, the diagnostic criteria were based on the descriptions in the NHANES: (1) No: no work activities; (2) Mild strength: causing small increases in respiration or heart rate; (3) Medium to high strength: causing large increases in brespiration or heart rate or the above situation exists. Hypertension was diagnosed based on self-reported history, taking anti-hypertensive medication, or blood pressure values were within the hypertension range[26]. Diabetes was diagnosed if a physician indicates that a person has diabetes, blood glucose test suggesting diabetes, or taking diabetes medication[27].”

2. Section “Results”: Please list incidence rates or estimates when referring to the risk for clinical outcomes. P-values alone do not provide any useful information.

**RESPONSE**: We appreciate your constructive and careful suggestions. We strongly agree with your suggestion and have added a description of mortality incidence in Table 3/4 and Supplementary Table S3.

**Table 3**. Multivariate adjusted Cox models for SII and all-cause mortality.

|  | Crude Model HR (95%CI) *P* | Fully adjusted Model HR (95%CI) *P* |
| --- | --- | --- |
| SII (continuous) (mortality incidence: 464/5497) | 1.0006 (1.0004, 1.0008), **< 0.0001** | 1.0004 (1.0002, 1.0006), **< 0.0001** |
| SII tertile |  |  |
| T1 (129/1832) | 1.0 | 1.0 |
| T2 (127/1832) | 0.9561 (0.7483, 1.2216), 0.7193 | 0.9012 (0.6888, 1.1789), 0.4477 |
| T3 (208/1833) | 1.5648 (1.2560, 1.9495), **< 0.0001** | 1.3268 (1.0398, 1.6930), **0.0230** |

Abbreviations: BMI, body mass index; PIR, family income-to-poverty ratio. The fully adjusted model was adjusted for all important covariates in the univariate analysis including age, BMI, PIR, sex, ethnicity, marital status, education level, WC, ALT, total cholesterol, smoking, alcohol consumption, physical work, hypertension, and diabetes.

**Table 4**. Multivariate adjusted Cox models for PIV and all-cause mortality.

|  | Crude Model HR (95%CI), *P*-value | Fully adjusted Model HR (95%CI), *P*-value |
| --- | --- | --- |
| PIV (continuous) (mortality incidence: 464/5497) | 1.0006 (1.0005, 1.0008), **< 0.0001** | 1.0006 (1.0003, 1.0008), **0.0002** |
| PIV tertile |  |  |
| T1 (111/1832) | 1.0 | 1.0 |
| T2 (146/1832) | 1.3407 (1.0473, 1.7161), **0.0200** | 1.2377 (0.9421, 1.6260), 0.1256 |
| T3 (207/1833) | 1.9742 (1.5674, 2.4867), **< 0.0001** | 1.3212 (1.0188, 1.7134), **0.0357** |

Abbreviations: BMI, body mass index; PIR, family income-to-poverty ratio. The fully adjusted model was adjusted for all important covariates in the univariate analysis including age, BMI, PIR, sex, ethnicity, marital status, education level, WC, ALT, total cholesterol, smoking, alcohol consumption, physical work, hypertension, and diabetes.

**Table S3**. Multivariate Cox models for SII and PIV and cardiovascular mortality.

|  | Crude Model HR (95%CI), *P*-value | Fully adjusted Model HR (95%CI), *P*-value |
| --- | --- | --- |
| SII (continuous) (mortality incidence: 75/5497) | 1.0006 (1.0002, 1.0011), **0.0087** | 1.0006 (1.0001, 1.0011), **0.0293** |
| SII tertile |  |  |
| T1 (19/1832) | 1.0 | 1.0 |
| T2 (21/1832) | 1.0609 (0.5703, 1.9738), 0.8519 | 1.1194 (0.5635, 2.2238), 0.7473 |
| T3 (35/1833) | 1.7620 (1.0074, 3.0820), **0.0471** | 1.7462 (0.9308, 3.2757), 0.0824 |
| PIV (continuous) (mortality incidence: 75/5497) | 1.0006 (1.0002, 1.0010), **0.0040** | 1.0007 (1.0002, 1.0013), **0.0076** |
| PIV tertile |  |  |
| T1 (15/1832) | 1.0 | 1.0 |
| T2 (27/1832) | 1.8207 (0.9683, 3.4236), 0.0629 | 1.8704 (0.9189, 3.8072), 0.0842 |
| T3 (33/1833) | 2.3110 (1.2545, 4.2575), **0.0072** | 2.1350 (1.0729, 4.2485), **0.0307** |

The crude Model was a crude model with no adjustment, and the fully adjusted model adjusted for all significant covariates in the univariate analysis including age, PIR, total cholesterol, HDL- cholesterol, physical work, hypertension, and diabetes. Abbreviations: SII, systemic immune-inflammation index; PIV, pan-immune-inflammation value; PIR, family income-to-poverty ratio; HR, hazard ratio; 95% CI, 95% confidence interval.

3. Supplemental Tables: Uni- and multivariate analyses can be shown for multiple outcomes in one table.

**RESPONSE**: We appreciate your constructive and careful suggestions.We combined the respective tables of univariate and multivariate Cox regression analyses in the Supplementary Material in response to comments, see Supplementary file.

Thank you very much for your patience and sincere comments in reviewing the manuscript and giving us the opportunity to revise it. We have responded and revised the manuscript according to your comments one by one, and we believe that the quality of our manuscript will be significantly improved and suitable for publication.

**Response to reviewer 2**

Major Concerns
1.The term "U-shaped positive correlation" used in the manuscript is somewhat misleading. It implies that the correlation remains consistently positive, which is not typically the case with a U-shaped relationship, where the direction of the relationship changes. This terminology could cause confusion for readers. The authors should clarify the mathematical calculation underlying this term or explain how they arrived at this specific terminology. Alternatively, the authors could reference the "turning point" in the discussion to provide a more accurate description of the relationship. A clearer explanation will help avoid misunderstandings and ensure that the findings are accurately interpreted.

**RESPONSE**: We appreciate your constructive and careful suggestions.We very much apologize for any distress caused by our inaccurate descriptions. In fact, our original intent was that there might be a U-shaped association between SII and all-cause mortality in patients with FLD (a positive was added by mistake). In the original manuscript, this statement was derived primarily from the visual graphical features (presenting a U-shape), consistent with the description of previous NHANES-related studies (such as PMID:36817427, PMID:38057733). However, we realized that this claim was not supported by the underlying mathematical counts, and therefore removed the U-shaped association statement from the manuscript and uniformly replaced it with a “nonlinear association”. In addition, to provide relevant analytic support, we performed a threshold effect analysis of this association, suggesting that the association between SII and all-cause mortality in the FLD population existed only after the turning point, and thus suggesting that the previous term “U-shaped positive association” was inaccurate. Therefore, we uniformly changed the term to “nonlinear association” and added descriptions and analytical support in the manuscript and supplementary materials. We replaced all descriptions in the discussion with non-linear correlations and “turning points”, as you suggested. As shown below: “Finally, we explored the threshold effect of the nonlinear association of SII with all-cause mortality in patients with FLD.”, “Threshold effect analysis showed a positive association (HR of 1.001) between SII and all-cause mortality in the FLD population after the turning point (SII=458.71) and a loss of association before the turning point, suggesting the presence of a threshold effect (**Table S6**).”

**Table S6**. Threshold effects analysis of the association of SII with all-cause mortality in the FLD population.

| **ALL** | SII<=458.71 | SII >458.71 | P for interaction |
| --- | --- | --- | --- |
| SII | 0.998 (0.997, 1.000) 0.05854 | 1.001 (1.000, 1.001) <0.00001 | <0.0001 |

The fully adjusted model was adjusted for all important covariates in the univariate analysis including age, BMI, PIR, sex, ethnicity, marital status, education level, WC, ALT, total cholesterol, smoking, alcohol consumption, physical work, hypertension, and diabetes.

2. The exclusion of patients who died within 24 months may introduce bias, as it could exclude individuals with the most severe disease. This exclusion criterion should be better justified or re-evaluated. In addition, the authors explain, that “we excluded extreme values of SI markers (N=70)“. Can the authors elaborate what they mean by that? How was “extreme” defined? Which cut offs were used and why? Excluding values of SI markers could also introduce bias, as it might remove data points that could be crucial for understanding the full range of the relationship between SI markers and mortality.

**RESPONSE**: We appreciate your constructive and careful suggestions.We strongly agree with your comments and recognize that these exclusions may introduce bias. In the previous manuscript, these descriptions were derived from previous NHANES-related studies (e.g. PMID: 36895572; PMID: 36172591), which may have been designed to exclude the effects of SI extremes (which may have originated from measurement anomalies) and short-term follow-up (which may have led to reverse causal associations). We performed sensitivity analyses without excluding these populations based on your comments and demonstrated that they did not significantly alter the results. Therefore, we believe that this exclusion did not significantly affect the conclusions drawn. See supplement to the manuscript and supplementary material: “Second, considering the potential bias introduced by excluding data with follow-up length <24 months and extreme data on SI markers, we did not exclude this group of patients in the sensitivity analysis to verify the stability of the conclusions.”, “Similar results were obtained by not excluding the population with a length of follow-up <24 months and extreme values of SI markers, i.e., SII/PIV remained significantly and positively associated with all-cause and CVD mortality in patients with FLD, confirming the stability of the conclusions (**Table S7 and S8**).”We believe that these analyses and explanations address your concerns and further strengthen the conclusions of the study. Thank you for your valuable suggestions, and please refer to the revised manuscript and supplementary materials for detailed modifications.

**Table S7**. Sensitivity analyses of the association of SII/PIV with all-cause mortality in patients with FLD (excluding those with <24 months of follow-up and extreme values of SI markers).

| **ALL** | **Fully adjusted Model HR (95%CI)** | **p** |
| --- | --- | --- |
| **SII** | 1.0006(1.0003,1.0009) | <0.001 |
| **SII** |  |  |
| T1 | ref | ref |
| T2 | 0.7468(0.5508,1.0126) | 0.0602 |
| T3 | 1.2792(0.8846,1.8499) | 0.1907 |
| **PIV** | 1.0009(1.0005,1.0013) | **<0.0001** |
| **PIV** |  |  |
| T1 | ref | ref |
| T2 | 1.1202(0.8002,1.5681) | 0.5085 |
| T3 | 1.4029(0.9815,2.0053) | 0.0632 |

The fully adjusted model was adjusted for all important covariates in the univariate analysis including age, BMI, PIR, sex, ethnicity, marital status, education level, WC, ALT, total cholesterol, smoking, alcohol consumption, physical work, hypertension, and diabetes.

**Table S8**. Sensitivity analyses of the association of SII/PIV with CVD mortality in patients with FLD (excluding those with <24 months of follow-up and extreme values of SI markers).

| **CVD** | **Fully adjusted model**  **HR (95%CI)** | **p** |
| --- | --- | --- |
| **SII** | 1.0009(1.0006,1.0013) | **<0.0001** |
| **SII** |  |  |
| T1 | ref | ref |
| T2 | 0.5549(0.3478,0.8852) | **0.0135** |
| T3 | 1.6423(1.0142,2.6596) | **0.0437** |
| **PIV** | 1.0011(1.0008,1.0014) | **<0.0001** |
| **PIV** |  |  |
| T1 | ref | ref |
| T2 | 0.9571(0.5238,1.7490) | 0.8867 |
| T3 | 2.2793(1.3660,3.8032) | **0.0016** |

The fully adjusted model was adjusted for all significant covariates including age, PIR, total cholesterol, HDL- cholesterol, physical work, hypertension, and diabetes.

3. The manuscript employs different approaches to adjusting for covariates in all-cause and cardiovascular mortality models. While age is a critical risk factor for mortality, it appears that age was not consistently adjusted for in the all-cause mortality models. This inconsistency can introduce significant bias and affect the validity of the study's conclusions. The authors should either provide a strong justification for this approach or revise their models to ensure that age is consistently adjusted for across all mortality analyses. This is crucial to maintain the methodological integrity of the study and the accuracy of its findings.

Building up on the previous comment, the authors demonstrate significant differences in possibly confounding factors such as age, BMI, Sex, and others across groups. These factors should be consistently adjusted for in the statistical models to ensure accurate results. If these covariates were not included in all models, the authors should provide a better explanation of how they selected the covariates for adjustment. The process of covariate selection is not clear to this reviewer, and without proper justification, there is a risk of introducing bias or misinterpreting the relationships between variables.

**RESPONSE**: We appreciate your constructive and careful suggestions.We strongly agree with your comments. In the previous manuscript, we constructed three Cox regression models, Model 1 ( crude model), Model 2 (partially adjusted), and Model 3 (fully adjusted model). This approach is informed by the practice in most previous NHANES-related studies (e.g., PMID: 36817427) of adjusting incrementally to see if these associations are independent of the covariates adjusted in each model. Consistent with previous NHANES-related studies, these covariates were selected with no specific required means, and generally the most common underlying covariates (e.g., demographic variables) were selected for initial adjustment to explore whether these associations were independent of the underlying demographic factors. We recognized the potential impact of not adjusting for important covariates in all models; therefore, we deleted the original partially adjusted model2 and retained only the crude and fully adjusted models (see the corresponding sections and tables in the manuscript). In the crude model, we did not adjust for any covariates and aimed to initially characterize the crude associations of these SI markers with mortality in the FLD population for comparison with the effect values and significance of the associations in the full-adjusted model. In the fully adjusted model, we fully adjusted for covariates that were significant in the univariate analyses to fully account for the effects of confounding factors. We believe this approach reduces these concerns and emphasizes that the true independent associations (in contrast to the crude model) are those in the fully adjusted model.

4. The retrospective design inherently limits the ability to infer causality. While this is common in cohort studies, the discussion could better address how this limitation might impact the findings.

**RESPONSE**: We appreciate your constructive and careful suggestions.We have added this part of the description to the discussion as per your comments,, as follows: “Therefore, these findings may only indicate an association between these SI markers and mortality in patients with FLD, rather than suggesting that SI directly contributes to increased mortality and needs to be interpreted with caution.”In addition, we have further discussed how the retrospective design may affect the study. This design may fail to capture all potential confounding factors, particularly those that could not be adequately controlled during follow-up. Therefore, although we observed a significant association between SI markers and mortality, these associations should not be interpreted as causal. Nevertheless, the findings still hold significant clinical value, as they lay the groundwork for future prospective studies and suggest that SI markers may play a key role in the prognosis of patients with FLD.We believe that these additions provide a more comprehensive explanation of the limitations of the retrospective design and offer clearer guidance for interpreting the study results. Thank you for your valuable suggestion.

5.The study relies heavily on NHANES data, which, while comprehensive, limits the analysis to the diagnostic tools and variables available within that dataset. The discussion could benefit from considering how additional diagnostic tools, might enhance future research.

**RESPONSE**: We appreciate your constructive and careful suggestions.We have added this part of the description to the discussion as per your comments, as follows: “Due to the data limitations of NHANES, future studies need to assess FLD using more accurate methods such as transient elastography to accurately reflect these associations.”Thank you again for your valuable suggestion.

6.The present study uses the Fatty Liver Index (FLI) as a diagnostic tool for FLD rather than liver biopsy, which may reduce diagnostic accuracy. While the authors acknowledge this limitation, they could have provided more justification or compared the FLI's diagnostic reliability with other methods in the discussion.

**RESPONSE**: We appreciate your constructive and careful suggestions.We have added this part of the description to the discussion as per your comments, as follows: “Liver biopsy, being invasive and subject to potential sampling errors and lack of inter-rater reliability, is impractical to conduct in large epidemiologic surveys[59]. In addition, a recent meta-analysis demonstrated that FLI had the highest predictive ability for hepatic steatosis compared to other noninvasive markers[60].”

References

59. Tincopa MA, Loomba R. Non-invasive diagnosis and monitoring of non-alcoholic fatty liver disease and non-alcoholic steatohepatitis. Lancet Gastroenterol Hepatol. 2023;8(7):660-70. doi: 10.1016/s2468-1253(23)00066-3.

60. Contreras D, González-Rocha A, Clark P, Barquera S, Denova-Gutiérrez E. Diagnostic accuracy of blood biomarkers and non-invasive scores for the diagnosis of NAFLD and NASH: Systematic review and meta-analysis. Ann Hepatol. 2023;28(1):100873. doi: 10.1016/j.aohep.2022.100873.

7.The manuscript introduces SIRI as one of the systemic inflammation markers but then downplays its relevance due to non-significant findings. This could be better integrated into the discussion by exploring why SIRI might not have shown significant associations, contrasting with SII and PIV.

**RESPONSE**: We appreciate your constructive and careful suggestions.We have added this part of the description to the discussion as per your comments, as follows: “Available evidence to explain the missing association of SIRI with mortality in patients with FLD is still lacking. Since SIRI differs from the two remaining SI markers mainly by not considering the effect of platelets, we speculate that platelet counts may have important prognostic value in patients with FLD. Interestingly, a recent meta-analysis showed that reduced platelet counts were associated with increased NAFLD severity[58].”In future studies, further exploration of the role of platelets in patients with FLD may help better understand why SIRI did not show an association with mortality. We also suggest that future research employ more advanced analytical methods to evaluate the role of SIRI and other systemic inflammation markers at different stages of disease progression.

References

58. Li L, Yu J, Zhou Z. Association between platelet indices and non-alcoholic fatty liver disease: a systematic review and meta-analysis. Rev Esp Enferm Dig. 2024;116(5):264-73. doi: 10.17235/reed.2022.9142/2022.

8.The U-shaped association between SII and mortality is intriguing but not fully explored in the discussion. The manuscript could expand on potential biological mechanisms underlying this relationship, offering more than just statistical interpretation.

**RESPONSE**: We appreciate your constructive and careful suggestions.We have added this part of the description to the discussion as per your comments, as follows: “Recent cohort studies similarly utilizing NHANES have shown that SII is associated with mortality in chronic kidney disease[42], osteoarthritis[43], and diabetes[44] populations. Most of the previous studies have similarly demonstrated nonlinear associations (e.g., U-shaped associations) between SII and clinical outcomes. These results may suggest that there is a threshold effect for these associations, i.e., only excessive SI promotes disease progression in FLD. Mild inflammatory responses may exert hepatoprotective effects by promoting tissue repair and homeostatic reconstruction[45]. However, the relevant biological mechanisms explaining why a certain range of SII is negatively associated with all-cause mortality in patients with FLD remain unknown.”

References

42. Huang P, Mai Y, Zhao J, Yi Y, Wen Y. Association of systemic immune-inflammation index and systemic inflammation response index with chronic kidney disease: observational study of 40,937 adults. Inflamm Res. 2024;73(4):655-67. doi: 10.1007/s00011-024-01861-0.

43. Zhou E, Wu J, Zhou X, Yin Y. Systemic inflammatory biomarkers are novel predictors of all-cause and cardiovascular mortality in individuals with osteoarthritis: a prospective cohort study using data from the NHANES. BMC Public Health. 2024;24(1):1586. doi: 10.1186/s12889-024-19105-5.

44. Zhang J, Fan X, Xu Y, Wang K, Xu T, Han T, et al. Association between inflammatory biomarkers and mortality in individuals with type 2 diabetes: NHANES 2005-2018. Diabetes Res Clin Pract. 2024;209:111575. doi: 10.1016/j.diabres.2024.111575.

45. Brenner C, Galluzzi L, Kepp O, Kroemer G. Decoding cell death signals in liver inflammation. J Hepatol. 2013;59(3):583-94. doi: 10.1016/j.jhep.2013.03.033.

9.Some figures, particularly the Kaplan-Meier curves and restricted cubic splines, are essential to understanding the findings. However, the figure legends provided in the manuscript are too brief, lacking detailed explanations. Each figure, especially those with multiple panels marked by letters (e.g., A, B, C, etc.), should include a comprehensive description. This should detail the meaning of each axis if abbreviations are used, and the statistical tests employed. Providing this information will enhance the clarity of the figures and ensure that readers can fully understand the visual data without needing to reference the main text.

**RESPONSE**: We appreciate your constructive and careful suggestions.We have added details to the legend of the figure based on comments to better illustrate the figure as follows:

**Figure 1**. Study subject inclusion flowchart, NHANES 2007-2018.

**Figure** **2**. KM models for SII and PIV with all-cause and cardiovascular mortality in patients with FLD, NHANES 2007-2018. A: KM plot (all-cause) for SII; B: KM plot (all-cause) for SIRI; C: KM plot (CVD-cause) for SII; D: KM plot (CVD-cause) for PIV. The horizontal axis of the KM plot represents survival time (months) and the vertical axis represents the corresponding (all-cause or CVD) survival probability. The log-rank test was used to analyze differences between groups.

**Figure 3**. RCS models of SII (A) and PIV (B) with all-cause mortality in patients with FLD, NHANES 2007-2018. The horizontal axis of the RCS plot represents the level of SII/PIV, and the vertical axis represents the log hazard value for all-cause mortality. In Panel A, the point where the two dashed lines cross is the turning point (458.71). RCS, restricted cubic splines.

**Figure 4**. Subgroup analysis of SII (A) and PIV (B) with all-cause mortality in patients with FLD, NHANES 2007-2018. Abbreviations: BMI, body mass index; PIR, family income-to-poverty ratio; ALT, alanine transaminase; T1, T2, T3 = tertile1, tertile2, tertile3.

Minor Concerns
10. The abstract and introduction sections are well-written but could be slightly condensed to improve focus. The statement "Future clinical research is needed to confirm these findings in diverse populations and with different diagnostic tools like transient elastography" is quite general and does not add specific value to the abstract. It is recommended to remove this statement to maintain the focus and precision of the abstract.

**RESPONSE**: We appreciate your constructive and careful suggestions.We have removed the “Future clinical research is needed to confirm these findings in diverse populations and with different diagnostic tools like transient elastography” section of the abstract at your suggestion to better focus on the content of the manuscript.

11. While the manuscript is generally well-cited, a few statements, particularly those regarding the general prognostic value of SII and PIV in other diseases, could benefit from additional references or more current studies

**RESPONSE**: We appreciate your constructive and careful suggestions.We have added the appropriate descriptions and added the most recent literature to our discussion as follows: “Recent cohort studies similarly utilizing NHANES have shown that SII is associated with mortality in chronic kidney disease[42], osteoarthritis[43], and diabetes[44] populations.”, “Furthermore, PIV has been widely implicated ..., sepsis patients[49], patients with ST elevation myocardial infarction[50], and patients with acute heart failure[51].”

References

42. Huang P, Mai Y, Zhao J, Yi Y, Wen Y. Association of systemic immune-inflammation index and systemic inflammation response index with chronic kidney disease: observational study of 40,937 adults. Inflamm Res. 2024;73(4):655-67. doi: 10.1007/s00011-024-01861-0.

43. Zhou E, Wu J, Zhou X, Yin Y. Systemic inflammatory biomarkers are novel predictors of all-cause and cardiovascular mortality in individuals with osteoarthritis: a prospective cohort study using data from the NHANES. BMC Public Health. 2024;24(1):1586. doi: 10.1186/s12889-024-19105-5.

44. Zhang J, Fan X, Xu Y, Wang K, Xu T, Han T, et al. Association between inflammatory biomarkers and mortality in individuals with type 2 diabetes: NHANES 2005-2018. Diabetes Res Clin Pract. 2024;209:111575. doi: 10.1016/j.diabres.2024.111575.

49. Xu HB, Xu YH, He Y, Lin XH, Suo Z, Shu H, et al. Association between admission pan-immune-inflammation value and short-term mortality in septic patients: a retrospective cohort study. Sci Rep. 2024;14(1):15205. doi: 10.1038/s41598-024-66142-6.

50. Murat B, Murat S, Ozgeyik M, Bilgin M. Comparison of pan-immune-inflammation value with other inflammation markers of long-term survival after ST-segment elevation myocardial infarction. Eur J Clin Invest. 2023;53(1):e13872. doi: 10.1111/eci.13872.

51. Inan D, Erdogan A, Pay L, Genc D, Demırtola AI, Yıldız U, et al. The prognostic impact of inflammation in patients with decompensated acute heart failure, as assessed using the pan-immune inflammation value (PIV). Scand J Clin Lab Invest. 2023;83(6):371-8. doi: 10.1080/00365513.2023.2233890.

12.• Both the terms "gender" and "sex" are used inconsistently throughout the manuscript, which can lead to confusion. To ensure clarity and scientific accuracy, it is important to standardize the terminology. If referring to biological differences, "sex" should be used consistently throughout the manuscript. Ensuring uniformity in terminology will improve the overall coherence and precision of the text.

**RESPONSE**: We appreciate your constructive and careful suggestions.In response to your comments, we have replaced all descriptions of “gender” in the manuscript with “sex” to more clearly reflect the purpose of the manuscript.

Thank you very much for your patience and sincere comments in reviewing the manuscript and giving us the opportunity to revise it. We have responded and revised the manuscript according to your comments one by one, and we believe that the quality of our manuscript will be significantly improved and suitable for publication.

Your sincerely,

All authors
