# Supplementary material for: Chronic systemic inflammation predicts long-term mortality among patients with fatty liver disease: Data from the National Health and Nutrition Examination Survey 2007–2018
Source: PLoS One. 2024 Nov 18;19(11):e0312877. doi: 10.1371/journal.pone.0312877 (PMC11573152; doi:10.1371/journal.pone.0312877)
Supplement: Attachment — Submitted filename: Response to reviewers.docx [file pone.0312877.s013.docx]

Dear Editors and Reviewers,

We sincerely appreciate your time, effort, and valuable feedback in reviewing our manuscript. Your thoughtful comments have given us the opportunity to improve our work, and we have carefully addressed each of the reviewers' suggestions. We believe that the revisions have significantly enhanced the quality of the manuscript. Please find below our detailed responses to the reviewers' comments.

Thank you once again for your guidance and consideration.

**Response to reviewer 1**

Major comments:
1. Section “Abstract”: While the author's effort is appreciated, the newly added content is way too long for the abstract. My initial comment only asks for more clear wording related to the survival analyses, not for a comprehensive summary of statistical methods (this should be described in the “Methods” section). Since there is no formal need to name all methods used in detail, it would be sufficient (instead of the newly included language) to state that the associations between chronic systemic inflammation and long-term mortality were analyzed (following the description of the study design). Please streamline the “Methods” section in the abstract accordingly.

**RESPONSE**: We appreciate your constructive and careful suggestions.We strongly agree with your comments and have streamlined the “Methods” section of the abstract in line with the suggestions, and the revised Methods section was as follows: “**Methods**: This was a retrospective cohort study. We included adult patients with FLD with complete data and analyzed the association between chronic SI and long-term mortality in patients with FLD. Systemic immune-inflammation index (SII), pan-immune-inflammation value (PIV), and systemic inflammation response index (SIRI) were evaluated based on peripheral blood counts and FLD was determined by the Fatty Liver Index.”.

2. Section “Methods”, paragraph “Participants”: This section should be renamed to something like “Study design”. Accordingly, the authors should only provide a description of the overall NHANES cohort, and inclusion and exclusion criteria for the present study. The study flow should be moved to “Results”, where it should be listed in a paragraph labeled “Participants”. Please also add the median duration of follow-up.

**RESPONSE**: We appreciate your constructive and careful suggestions.We strongly agree with your comments and have made one-to-one modifications in accordance with your modification instructions. We added a description of the follow-up duration to the “Participants” section of the results as requested. The corresponding sections of the modified methods and results were as follows:

**METHODS**

**Study design**

NHANES is a nationally representative, multistage, cross-sectional survey conducted by the National Center for Health Statistics (NCHS) and is designed to assess the health and nutrition status of US non-institutionalized populations. The NCHS Ethics Review Board approved all NHANES study protocols, and all participants provided written informed consent. We utilized data from NHANES 2007-2018 (including 6 continuous cycles, 2007-2008, 2009-2010, 2011-2012, 2013-2014, 2015-2016, 2017-2018) for this study. Baseline FLD participants from NHANES 2007-2018 were followed until December 31, 2019, and mortality data was obtained by prospectively matching with death certificate information from the Public-use Linked Mortality Files of the National Death Index (NDI) database (https://www.cdc.gov/nchs/data-linkage/mortality-public.htm)[13, 18]. The study inclusion criteria included FLD patients aged 18 years or older with complete data. Exclusion criteria were participants with missing data and extreme SI values and a follow-up duration of less than 24 months.

**RESULTS**

**Participants**

We initially enrolled 57,414 individuals aged 18 years and older and excluded those without diagnostic indicators for FLD (N=40,125). Next, we excluded individuals with missing mortality data (N=2325), missing blood biochemical tests data (N=54), missing covariate data such as marital status (N=721), education level (N=14), hypertension (N=137), smoking (N=12), and physical work (N=2). Finally, from the population with complete data, we excluded extreme values of SI markers (N=70), those without FLD according to the diagnostic criteria (N=7832), and patients who died within 24 months of follow-up (N=626). Therefore, 5497 patients with FLD (2954 men and 2543 women) were included in the current study. (Figure 1) The median follow-up duration for FLD patients was 88 months(interquartile range: 55-121 months).

3. Section “Results”, “Baseline characteristics”: Since you are comparing data across groups of increasing inflammatory burden - please replace the current p-values (based on the methods paragraph, the present numbers seem to be overall-p-values) with p-values for trend. Please also indicate in the heading of the column that p-values for trends are used.

**RESPONSE**: We appreciate your constructive and careful suggestions.We strongly agree with your comment and have changed the original p-value to “p-values for trends” in the corresponding section of the results and table 1/2. Please see the corresponding revised section of the manuscript.

4. Section “Results”, “Tables 3+4”: In the current version, the newly added event numbers are not clearly labeled and added in the first column (which should only list the variables). Please add a separate column for the newly incorporated event numbers, and please also make sure to include an appropriate heading.

**RESPONSE**: We appreciate your constructive and careful suggestions.We strongly agree with your comments and have added an additional column to Table 3/4 to describe the mortality incidence overall and in the different tertiles. Please see revised table 3/4 below:

**Table 3**. Multivariate adjusted Cox models for SII and all-cause mortality.

|  | Mortality incidence | Crude Model HR (95%CI) *P* | Adjusted Model HR (95%CI) *P* |
| --- | --- | --- | --- |
| SII (continuous) | 464/5497 | 1.0006 (1.0004, 1.0008), **< 0.0001** | 1.0004 (1.0002, 1.0006), **< 0.0001** |
| SII tertile |  |  |  |
| T1 | 129/1832 | 1.0 | 1.0 |
| T2 | 127/1832 | 0.9561 (0.7483, 1.2216), 0.7193 | 0.9012 (0.6888, 1.1789), 0.4477 |
| T3 | 208/1833 | 1.5648 (1.2560, 1.9495), **< 0.0001** | 1.3268 (1.0398, 1.6930), **0.0230** |

Abbreviations: BMI, body mass index; PIR, family income-to-poverty ratio. The adjusted model was adjusted for all important covariates in the univariate analysis including age, BMI, PIR, sex, ethnicity, marital status, education level, WC, ALT, total cholesterol, smoking, alcohol consumption, physical work, hypertension, and diabetes.

**Table 4**. Multivariate adjusted Cox models for PIV and all-cause mortality.

|  | Mortality incidence | Crude Model HR (95%CI), *P*-value | Adjusted Model HR (95%CI), *P*-value |
| --- | --- | --- | --- |
| PIV (continuous) | 464/5497 | 1.0006 (1.0005, 1.0008), **< 0.0001** | 1.0006 (1.0003, 1.0008), **0.0002** |
| PIV tertile |  |  |  |
| T1 | 111/1832 | 1.0 | 1.0 |
| T2 | 146/1832 | 1.3407 (1.0473, 1.7161), **0.0200** | 1.2377 (0.9421, 1.6260), 0.1256 |
| T3 | 207/1833 | 1.9742 (1.5674, 2.4867), **< 0.0001** | 1.3212 (1.0188, 1.7134), **0.0357** |

Abbreviations: BMI, body mass index; PIR, family income-to-poverty ratio. The adjusted model was adjusted for all important covariates in the univariate analysis including age, BMI, PIR, sex, ethnicity, marital status, education level, WC, ALT, total cholesterol, smoking, alcohol consumption, physical work, hypertension, and diabetes.

5. Section“Results”: Despite my previous comment, the results section and its headings are still structured based on the statistical methods used. In this current version, it is hard to follow the logic. To improve the structure, please organize the sections in a way that is consistent with common standards for the reporting of clinical research. Regarding this manuscript, use sections such as participants, baseline characteristics, categorical and continuous associations between markers of chronic systemic inflammation and long-term mortality… In other words, the logic of reporting should be organized based on paragraphs related to findings, not according to the methods. Please structure the section and its headings accordingly.

**RESPONSE**: We appreciate your constructive and careful suggestions.We strongly agree with your suggestions and apologize for the unsuitability of the previous revisions. In response to your comments, we have changed the headings of the Results section accordingly to reflect the specific content of the findings. Please see section headings below:

**Participants**

We initially enrolled 57,414 individuals aged 18 years and older and excluded those without diagnostic indicators for FLD (N=40,125). Next, we excluded individuals with missing mortality data (N=2325), missing blood biochemical tests data (N=54), missing covariate data such as marital status (N=721), education level (N=14), hypertension (N=137), smoking (N=12), and physical work (N=2). Finally, from the population with complete data, we excluded extreme values of SI markers (N=70), those without FLD according to the diagnostic criteria (N=7832), and patients who died within 24 months of follow-up (N=626). Therefore, 5497 patients with FLD (2954 men and 2543 women) were included in the current study. (**Figure 1**) The median follow-up duration for FLD patients was 88 months(interquartile range: 55-121 months).

**Baseline characteristics**

**Table 1** described the baseline characteristics of patients grouped according to SII tertiles (≤ 370.9, 370.9 ~ 571.8, and ≥ 571.8). BMI, WC, ALT, AST, SII, PIV, SIRI, sex, and ethnicity showed significant differences between groups (all p-values for trend < 0.0001). Statistically significant trends were also observed for total cholesterol, hypertension, diabetes, and smoking (all p-values for trend < 0.05). However, no significant differences were observed between groups in age, PIR, HDL-cholesterol, marital status, education level, alcohol consumption, and physical work (all p-values for trend > 0.05). **Table 2** presented the baseline characteristics of patients grouped according to PIV tertiles (≤ 184.4, 184.4 ~ 313.8, and ≥ 313.8). Significant differences were observed in BMI, SII, PIV, SIRI, ethnicity, and smoking (all p-values for trend < 0.0001), and in age, ALT, total cholesterol, HDL-cholesterol, education, hypertension, and diabetes between groups (all p-values for trend < 0.05). However, no significant differences were found for PIR, AST, sex, marital status, alcohol consumption, and physical work (all p-values for trend > 0.05). Baseline analysis grouped by tertile of SIRI was presented in **Table S1**.

**Continuous and categorical associations of SI markers with long-term mortality in FLD**

A total of 464 all-cause deaths and 75 cardiovascular deaths were recorded during the follow-up period. Univariate Cox regression indicated that age, BMI, PIR, WC, ALT, total cholesterol, SII, PIV, male sex (with female sex as reference), non-Hispanic Black and non-Hispanic White in ethnicity (with Mexican American as reference), married, education > high school (with < high school as reference), presence of hypertension, presence of diabetes, previous and current alcohol consumption, previous and current smokers, and mild and medium-high physical work were associated with all-cause mortality in patients with FLD; for cardiovascular mortality, age, PIR, total cholesterol, HDL-cholesterol, SII, PIV, presence of hypertension, presence of diabetes, and medium-to-high physical work were significantly associated. Consistently, SIRI was not associated in either all-cause or cardiovascular mortality, and was therefore excluded from further discussion in the multivariate analysis. (**Table S2**)

Multivariate Cox proportional hazards regression analysis demonstrated that SII and PIV (continuous variable) both were significantly associated with all-cause mortality in the adjusted models (HR 1.0004, *P* < 0.0001 for SII and 1.0006, *P* = 0.0002 for PIV). When SII and PIV were treated as categorical variables, both SII and PIV in tertile 3 of the adjusted models (with tertile 1 as the reference) demonstrated significant relevance for all-cause mortality (adjusted model: HR 1.3268, *P* = 0.0230 for SII and HR 1.3212, *P* = 0.0357 for PIV). (**Table 3 and 4**) Similarly, both SII and PIV were significantly correlated with CVD mortality when treated as continuous variables; however, when treated as categorical variables, PIV (but not SII) was significantly associated with CVD mortality in the adjusted model (HR 2.1350, *P* = 0.0307 for PIV and 1.7462, *P* = 0.0824 for SII). (**Table S3**)

**Association of SII and PIV with all-cause and CVD-related survival probability in FLD**

The KM analysis showed that FLD patients with high tertiles of SII and PIV (compared to low tertiles) both presented lower all-cause related survival (both adjusted *P* values < 0.0001). However, PIV, but not SII based on tertile was significantly associated with the survival probability from CVD in patients with FLD, with higher PIV having a lower survival probability (adjusted *P* = 0.023), while a similar trend was observed for SII (adjusted *P* = 0.069). (**Figure 2**)

**Potential nonlinear correlation investigation**

RCS analysis demonstrated a nonlinear association between SII and all-cause mortality in patients with FLD (turning point = 458.71, *P* non-linear = 0.0032), while linear correlation was observed between PIV and all-cause mortality (*P* = 0.2744). (**Figure 3**) Both SII and PIV had a linear correlation with cardiovascular mortality among patients with FLD (both *P* non-linearity > 0.05). (**Figure S1**)

**Association of SII and PIV with mortality in FLD across subgroups**

The *P* for interaction values in all adjusted stratified analyses were > 0.05, suggesting that for SII and PIV, the association with all-cause mortality did not vary across subgroups. (**Figure 4**) Similarly, for cardiovascular mortality, the *P* for interaction values were > 0.05 across all subgroups. (**Figure S2**)

**Interactions with other inflammatory markers, threshold effects, and stability of findings**

CRP was similarly positively associated with all-cause and CVD mortality in the FLD population (all-cause: HR 1.089, p=0.0314; CVD: HR 1.122, *P*=0.0360). Notably, a significant positive correlation was also observed between SII/PIV and CRP (both β 0.001), suggesting that SII/PIV and other inflammatory markers, such as CRP, may significantly interact and collectively be associated with mortality in the FLD population (**Table S4 and S5**). Threshold effect analysis showed a positive association (HR 1.001) between SII and all-cause mortality in the FLD population after the turning point (SII=458.71) and a loss of association before the turning point, suggesting the presence of a threshold effect (**Table S6**). Similar results were obtained when the population with a follow-up period of less than 24 months and extreme SI marker values was not excluded, i.e., SII/PIV remained significantly and positively associated with all-cause and CVD mortality in patients with FLD, confirming the stability of the findings (**Table S7 and S8**).

Minor comments:
1. While the revised manuscript appears approved, it still contains multiple sections that are in parts hard to read due to grammar issues. Please revise the grammar and syntax of the whole manuscript to ensure appropriate readability. English language editing is highly recommended.

**RESPONSE**: We appreciate your constructive and careful suggestions.We strongly agree with your comments and have revised the full text for grammar and fluency. We invited specialized professionals to professionally edit the revised manuscript in the English language.

2. If only crude and one adjusted model are used, there is no need to call the adjusted model fully adjusted, since the is no other model adjusted for fewer covariates.

**RESPONSE**: We appreciate your constructive and careful suggestions.We strongly agree with your comments and have deleted “fully” before adjusted model throughout the text to accurately reflect the model adjustments.

3. The figure legends are in part not comprehensive enough. Please review and improve to ensure a clear wording.

**RESPONSE**: We appreciate your constructive and careful suggestions.We strongly agree with your comments and have added and improved the figure legend. The revised figure legend was as follows:

**Figure legend**

**Figure 1**. Study subject inclusion and exclusion flowchart, NHANES 2007-2018.

**Figure** **2**. KM models for SII and PIV with all-cause and cardiovascular mortality in patients with FLD, NHANES 2007-2018. A: KM plot (all-cause) for SII; B: KM plot (all-cause) for SIRI; C: KM plot (CVD-cause) for SII; D: KM plot (CVD-cause) for PIV. The horizontal axis of the KM plot represents survival time (months) and the vertical axis represents the corresponding (all-cause or CVD) survival probability. The log-rank test was used to analyze differences between groups.The low (T1), middle (T2), and high (T3) tertiles of the SII/PIV were indicated using red, green, and blue solid lines, respectively. Abbreviations: KM, Kaplan-Meier; FLD, fatty liver disease; CVD, cardiovascular disease.

**Figure 3**. RCS models of SII (A) and PIV (B) with all-cause mortality in patients with FLD, NHANES 2007-2018. The horizontal axis of the RCS plot represents the level of SII/PIV, and the vertical axis represents the log hazard value for all-cause mortality. In Panel A, the point where the two dashed lines cross is the turning point (458.71). In panel A, the p non-linear of 0.0032 represented the presence of a non-linear association between SII and all-cause mortality in patients with FLD, while in panel B, the p non-linear was 0.2744, suggesting a linear correlation. Abbreviations: RCS, restricted cubic splines; FLD, fatty liver disease.

**Figure 4**. Subgroup analysis of SII (A) and PIV (B) with all-cause mortality in patients with FLD, NHANES 2007-2018. In subgroup analyses, “Characteristics” represented the different subgroups included, “Total (N)” represented the number of participants in each subgroup, “HR” represented the effect value of the association in each subgroup ( hazard ratio), “Adjusted *P* value” represented the significance of the associations in the subgroups, and “*P* for interaction” represented whether the associations were significantly different between the subgroups. Abbreviations: BMI, body mass index; PIR, family income-to-poverty ratio; ALT, alanine transaminase; T1, T2, T3 = tertile1, tertile2, tertile3.

Thank you very much for your patience and sincere comments in reviewing the manuscript and giving us the opportunity to revise it. We have responded and revised the manuscript according to your comments one by one, and we believe that the quality of our manuscript will be significantly improved and suitable for publication.
